# Highlights from the 58th Bürgenstock Conference on Stereochemistry 2025

**DOI:** 10.1039/d5sc90175h

**Published:** 2025-08-29

**Authors:** Mattia Silvi, Claudia Bonfio

**Affiliations:** a The GSK Carbon Neutral Laboratories for Sustainable Chemistry, University of Nottingham Nottingham NG7 2TU UK Mattia.silvi@nottingham.ac.uk; b School of Chemistry, University of Nottingham Nottingham NG7 2RD UK; c Department of Biochemistry, University of Cambridge Tennis Court Road CB2 1GA Cambridge UK cb2036@cam.ac.uk

## Abstract

Herein, we share an overview of the scientific highlights from speakers at the latest edition of the longstanding Bürgenstock Conference.
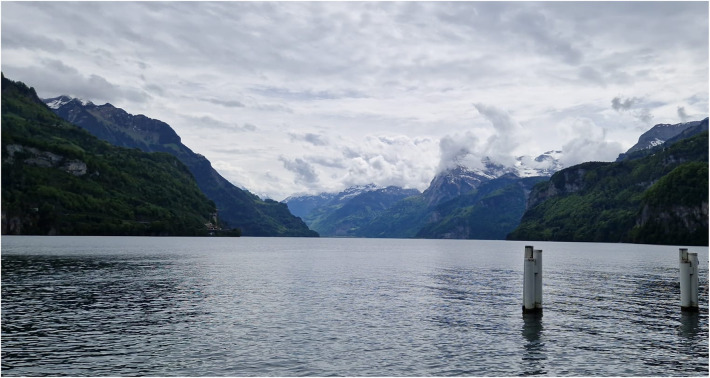

“Science is a social system… it has a system for communicating that knowledge… a compulsion if not an addiction for doing so.” – Roald Hoffmann, Nobel Prize interview, January 17, 2005.

## Introduction

The Bürgenstock Conference stands as a premier international forum in organic chemistry, renowned for its exceptional quality and global influence. Over the years, its scope has significantly evolved to foster an interdisciplinary environment, embracing fields such as organic synthesis, biological chemistry, and materials science. The convergence of leading speakers and high-profile attendees consistently generates inspiring discussions and a uniquely stimulating intellectual exchange.

The 58th Bürgenstock Conference was inaugurated by President José Luis Mascareñas, who underscored the event's commitment to scientific excellence and its remarkable diversity across topics, geographical representation of speakers, and gender balance. A warm introduction was also extended to the guest of honour, Professor Miquel Pericàs, acknowledging his outstanding scientific leadership and substantial contributions to the Spanish organic chemistry community, and more widely to the wider international scientific stage.

Despite the inclement weather the exceptional quality of the scientific program, encompassing talks, poster presentations, and engaging discussions, thoroughly captivated participants.

## David MacMillan – harnessing visible light in chemistry

Introduced by the conference vice-president Paolo Melchiorre, the event opened with an exceptionally inspiring keynote lecture by Nobel Laureate David MacMillan (Princeton University), marking his second appearance at the conference (his first being in 2003).

A defining strength of Professor MacMillan's research lies in his ability to contextualize innovative synthetic methodologies through impactful real-world applications, often facilitated by close collaborations with pharmaceutical stakeholders. His lecture began with the presentation of a cutting-edge microenvironment-mapping platform that leverages Dexter energy transfer-induced photocatalytic carbene generation for proximity labelling.^1^ This approach enables the investigation of intricate protein–protein interactions on cell membranes, offering promising avenues for addressing cancer types that are resistant to current therapies.

On a more synthetic front, Professor MacMillan showcased his powerful method for generating radicals from native alcohols^2^—one of the most abundant functional groups in nature—and their subsequent application in C–C bond formation. Notably, this includes the synthesis of valuable sp^3^-rich polycyclic frameworks from simple diols.^3^ He concluded with an exciting perspective on new strategies for sp^3^–sp^3^ couplings *via* selective radical cross-coupling, employing a cobalt-catalyzed homolytic substitution (S_H_2) mechanism. This approach holds tremendous potential, as demonstrated by its capacity to selectively combine radicals derived from widely available alcohols.^4^

## Claudia Höbartner – molecular architectures of RNA-alkylating ribozymes

Professor Claudia Höbartner (University of Würzburg) delivered an illuminating talk on the expanding functional landscape of RNA, exploring how chemical modifications can endow nucleic acids with new catalytic and recognition capabilities.

Her group's research reveals how nucleotide modifications, such as methylation and formylation, can be leveraged to design artificial ribozymes with precise site-selectivity. One highlight was a *de novo* methyltransferase ribozyme that methylates the N1 position of adenosine *via* general acid catalysis and active-site rigidification, mimicking natural RNA-modifying enzymes.^5,6^ Serendipitous discoveries also led to the development of a formylating ribozyme useful for fluorogenic labelling, as well as ribozymes capable of benzylation, a modification potentially relevant for protein tagging.

These studies suggest that RNA's chemical potential may once have extended far beyond its current biological roles. Looking forward, Professor Höbartner envisions harnessing synthetic cofactors, such as SAM analogues,^7^ to develop new bioconjugation tools and shed light on RNA's catalytic past.

## Andrey Klymchenko – chemistry of dyes at the nanoscale

Professor Andrey Klymchenko (CNRS, University of Strasbourg) delivered an inspiring lecture on the chemistry of fluorescent dyes and nanoparticles as advanced tools for biological imaging and diagnostics. His group tackles the limitations of traditional solvatochromic dyes, such as poor photostability and UV-range excitation, by engineering new, environmentally sensitive fluorophores that respond to local polarity and membrane dynamics.

A key focus lies in developing small, highly photostable dyes based on fluorene cores, with tailored push–pull electronic properties and organelle-specific targeting. These dyes can detect subtle differences in lipid composition or protein–lipid interactions by shifting their fluorescence based on the local environment, enabling sensitive mapping of biomembrane properties.^8,9^

Professor Klymchenko also introduced innovative supramolecular probes embedded in lipid nanoreactors for real-time sensing of bioactive amines. These systems exploit dynamic and covalent chemistries, such as reversible imine formation or irreversible pyrylium binding, to detect neurotransmitters like dopamine, without requiring biological receptors. This approach opens up new avenues for probing cellular chemistry with high specificity and minimal perturbation.^10^

## Jonathan Burton – oxonium ions in natural products and total synthesis

Introduced by Professor Karl Gademann, the conference continued with an inspiring lecture by Professor Jonathan Burton (University of Oxford), who shed light on the intriguing reactivity of oxonium ions. Although these species bear significant synthetic potential—*e.g.*, the well-known Meerwein reagent—they are often regarded as “exotic” and are seldom invoked as synthetic intermediates due to their fleeting nature under standard reaction conditions.

Through a compelling combination of low-temperature NMR spectroscopy and computational studies, Professor Burton explored the role of oxonium ions in the biosynthesis of a diverse family of natural products.^11–14^ His research not only elucidated the reactivity patterns of these often elusive intermediates—governed by orbital interactions, ring strain, and other structural factors—but also enabled the biomimetic synthesis of 25 natural products. The considerable insights presented promise to provide a rational framework for understanding biosynthetic pathways that proceed through common oxonium intermediates.

Overall, the findings suggest that oxonium ions may play a more widespread role in natural biosynthetic processes than previously recognized. Furthermore, leveraging these intermediates in synthetic strategies could unlock new possibilities in complex molecule construction and broaden the toolkit of modern organic synthesis.

## Rebecca Ruck – enhancing enabling technologies in industrial chemical synthesis

Introduced by Professor Karl Gademann, Rebecca Ruck delivered a compelling talk highlighting recent efforts at MSD to improve the sustainability and competitiveness of chemical manufacturing through a range of enabling technologies. Her lecture brought a valuable industrial perspective to the conference, enriching the diversity of the scientific discussion.

Dr Ruck began by illustrating the pivotal role of biocatalysis in accessing non-canonical amino acids, structures in which Merck has been interested for various synthesis endeavours, including accessing macrocyclic peptides.^15,16^ Notably, she presented the development of a single-step synthesis of 3-hydroxyproline *via* an engineered proline oxidase, as well as the use of a transaminase-based strategy to obtain β-branched amino acids.^17^ She then detailed the application of flow chemistry to solid-phase peptide synthesis (SPPS), a strategy that significantly enhances both time efficiency and process mass intensity in protein synthesis.

The presentation concluded with an integrated approach combining high-throughput experimentation (HTE) and flow chemistry to optimize the production of antibody–drug conjugates, showcasing how modern process development tools can streamline complex pharmaceutical manufacturing.

## Bert Weckhuysen – *operando* spectroscopy and insights in catalysis to develop the chemicals and the fuels of tomorrow

The third day of the Bürgenstock Conference opened with a compelling session focused on heterogeneous catalysis, introduced by Professor Gilles Gasser. The first speaker of the day, Professor Bert Weckhuysen from Utrecht University, posed a thought-provoking question inspired by his passion for photography: can we “photograph”—or even “film”—catalytic activity to better understand and improve catalytic processes?

This question is particularly relevant in the context of heterogeneous catalysis, where active sites are not static but dynamically fluctuate—their positions and characteristics changing over time across the catalyst surface. Professor Weckhuysen demonstrated how *operando* spectroscopy^18^ can provide real-time, molecular-level insight into these dynamic systems, paving the way for the development of more efficient and sustainable catalytic processes.

His lecture showcased ambitious research aimed at moving beyond traditional fossil-based feedstocks toward a crude oil-free refinery model.^19^ This includes catalytic strategies for converting plastic waste, biomass, and CO_2_ into valuable chemicals and fuels.^20,21^ Among the highlights were recent advances in the catalytic conversion of CO_2_ into methane and aromatics—achieved *via* a telescoped process—as well as the catalytic cracking of polymers to produce useful chemical building blocks.

## Diego Peña – merging surface science and organic chemistry for advanced materials

The second talk of the day was delivered by Professor Diego Peña (CiQUS), who presented innovative research at the interface of surface catalysis and homogeneous catalysis, aimed at developing advanced materials with broad applications in materials science.

His lecture focused particularly on the synthesis of novel nanographenes—polycyclic aromatic structures with promising properties for use as organic semiconductors. The general approach involves the synthesis of polyaromatic precursors using established synthetic strategies, including benzyne chemistry and cross-coupling methodologies.^22^ These precursors then undergo on-surface planarization, enabling the formation of nanographenes, which can be visualized and characterized at the molecular level using atomic force microscopy (AFM) or scanning tunneling microscopy (STM).^23^

Professor Peña showcased a range of applications for these materials. These include the development of organic semiconductors, structural elucidation of complex environmental pollutants such as jet fuel residues and asphaltenes,^24^ exploration of open-shell nanographene layers,^25^ and the synthesis of nanoporous graphene—an emerging semipermeable material with significant practical potential in filtration and separation technologies.

### Magic moments

True to its tradition of blending world-class science with moments of levity and connection, this year's Bürgenstock Conference delivered an unexpected and delightful surprise: a magic show. Departing from the usual concert format, President José Luis Mascareñas introduced an evening of illusion and laughter that captivated the entire audience. With charm and sleight of hand, occasionally involving the audience's own money, the magician created a playful atmosphere that had professors and participants alike in stitches. It was a light-hearted interlude that brought the community together in a new and memorable way, reminding us that a bit of magic has its place even in the most rigorous scientific settings.

## Anat Milo – harnessing data science and organocatalysis in synthesis

Introduced by Professor Olalla Vázquez, the morning session of the fourth day of the 58th Bürgenstock Conference began with an engaging and visually rich presentation by Professor Anat Milo (Ben-Gurion University of the Negev). She illustrated how the integration of small data analysis, data visualization, and human intuition can significantly enhance the selectivity and efficiency of chemical reactions.

Professor Milo showcased her recent success in expanding the scope of the asymmetric carbene-organocatalyzed benzoin reaction through the use of boronic acids. These acids form well-defined adducts with the catalyst–substrate complex, enabling the formation of highly geometrically defined intermediates and improved reaction outcomes.^26,27^

Further, she demonstrated how data visualization and analysis can guide chemists in optimizing reaction conditions, particularly in methodologies requiring systematic variation within reaction scope entries. This approach was exemplified by studies on isotope exchange reactions.^28^

Concluding her talk, Professor Milo discussed the promising potential of building accurate predictive models in chemistry by leveraging small data sets^29^ fed into existing foundation models. This strategy may pave the way toward precise predictions of molecular properties through targeted small data analysis.

## Eva Hevia – harnessing the potential of alkali metals in synthesis

After the coffee break, Professor Eva Hevia from the University of Bern delivered an exceptionally inspiring lecture on base metal catalysis. She presented a Schlosser superbase-inspired strategy that leverages the cooperative effects of potassium *tert*-butoxide (KO*t*Bu) and a sterically hindered zinc-amide base to achieve rapid zincation of aromatic compounds.^30^

Professor Hevia also discussed a practical system for the sodiation of aromatics and highlighted several valuable applications, including isotope exchange reactions, borylation, and an iron-catalyzed sp^2^–sp^3^ cross-coupling process.^31–33^ This latter example challenges the long-held notion that alkyl sodium species are difficult-to-control intermediates.

One of the most striking aspects of Professor Hevia's research is the seamless integration of practical applicability with deep mechanistic understanding. Her group has conducted extensive studies on the nature of the active catalytic species, often isolating and structurally characterizing them using X-ray diffraction techniques, thereby providing crucial insights into the underlying chemistry.

## Kathrin Lang – expanding the genetic code

Professor Kathrin Lang (ETH Zurich) presented a remarkable lecture on genetic code expansion as a platform for engineering proteins with bespoke chemical functionalities. By incorporating non-canonical amino acids (ncAAs) into proteins *via* orthogonal *t*RNA/synthetase pairs, her team creates powerful tools to visualise, interrogate, and manipulate complex biological processes.

She illustrated how site-specific incorporation of ncAAs enables bioorthogonal labelling and light-induced crosslinking to study protein–protein interactions in live cells. Other applications include installing post-translational modifications, such as succinylation, to investigate their regulatory roles in metabolism.^34^

Professor Lang further introduced novel strategies to decode ubiquitin signalling by generating defined ubiquitin topologies *via* proximity-guided sortylation. These synthetic “ubi-writers” could enable precise control over ubiquitination patterns, allowing for the study of proteasomal processing and cellular signalling pathways.^35,36^

Pushing boundaries even further, her group hijacked native peptide transporters to import tripeptides bearing ncAAs, enabling simultaneous dual labelling through amber and ochre suppression. Together, these innovations dramatically expand the chemical space of proteins, offering a molecular toolbox for probing and reprogramming biology with unprecedented precision.

## Talks and poster sessions

A hallmark of the Bürgenstock Conference is the exceptional quality and diversity of its short talks and poster sessions, which offer a stage for early-career researchers from both academia and industry to present cutting-edge science in an atmosphere of open, engaged exchange. This year's contributions spanned a remarkably interdisciplinary landscape, blending synthetic methodology, chemical biology, catalysis, materials science, and computational chemistry.

Attendees were treated to exciting developments in topics as varied as photoredox autocatalysis, sesquiterpene biosynthesis, benzyne chemistry, and cross dehydrogenative coupling, alongside innovative applications in nanopore sequencing (memorably illustrated using ping pong ball-chains and Pringles tubes). Other highlights included machine learning approaches to molecular design, site-selective fluorination strategies, chemically modified proteins, and iron-based photocatalysis.

The lively poster sessions created an ideal forum for in-depth discussion, often blurring the lines between disciplines and sectors. From evolving enzymes for biocatalysis to graphene-based materials and digital retrosynthetic tools, the presentations were uniformly characterised by scientific rigour, creativity, and ambition. These sessions captured the very spirit of this conference: a community of chemists pushing boundaries, exchanging ideas, and shaping the future of the field.

## Shu-Li You – innovations in asymmetric catalysis and dearomative functionalizations

The final session of the day was introduced by Professor Erick Carreira, who welcomed Professor Shu-Li You from the Shanghai Institute of Organic Chemistry (SIOC). In a stimulating presentation, Professor You highlighted his significant contributions to asymmetric catalysis, with a particular focus on dearomative functionalization reactions that enable access to enantiomerically enriched quaternary carbon centers.

His work employs a range of innovative asymmetric functionalization strategies, mainly involving phosphoric acid organocatalytic methods^37^ and transition metal catalysis^38^—primarily iridium- and rhodium-catalyzed allylation reactions. Professor You also showcased advances in photocatalysis^39^ before concluding with a highly practical synthetic strategy for the preparation of enantioenriched *Z*-alkenes, which are traditionally challenging to synthesize *via* conventional methods.^40^

This approach leverages the unique reactivity of iridium complexes, which stereospecifically interact with *Z*-allyl substrates to form configurationally stable π-allyl complexes resistant to isomerization. This enables a direct and efficient route to chiral, enantioenriched *Z*-alkenes, expanding the synthetic toolkit for these valuable motifs.

## Belén Martín-Matute – advances in homogeneous catalysis for late-stage C–H functionalization

The final day of the Bürgenstock Conference was introduced by Professor Francesca Paradisi and commenced with an inspiring lecture by Professor Belén Martín-Matute (Stockholm University), focusing on homogeneous catalysis strategies for C–H bond functionalization, with particular emphasis on applications in late-stage modification.

Professor Martín-Matute began by detailing a versatile and general iridium-catalyzed *ortho* C–H functionalization of benzoic acids, showcasing transformations including iodination,^41^ methylation,^42^ and amination.^43^ She further explored iridium catalysis in substitution reactions involving aliphatic alcohols, which serve as practical starting materials for amination processes.^44^

The lecture concluded with a demonstration of stereospecific 1,3-proton shift reactions that convert readily accessible allylic alcohols into synthetically valuable carbonyl compounds. This methodology can be leveraged to access chiral, enantioenriched molecules from corresponding enantioenriched starting materials, using a relatively bulky guanidine organic base to control stereochemistry.^45^

## Todd Hyster – photobiocatalysis for asymmetric radical reactions

The final lecture of the 2025 Bürgenstock Conference was introduced by Professor Francesca Paradisi and delivered by Professor Todd Hyster (Princeton University). In a compelling and forward-looking presentation, Professor Hyster illustrated how photobiocatalysis can offer exceptionally robust and selective synthetic routes to enantioenriched chiral compounds.

He described how redox-active enzyme cofactors can engage in electron donor–acceptor (EDA) complexes or cooperate with traditional photocatalysts to facilitate electron transfer processes. This powerful activation strategy enables the generation of open-shell intermediates which, when paired with the inherent chirality of enzymes, allows for highly enantioselective radical transformations—long-standing challenges in catalysis due to the typically low stereocontrol associated with radical pathways.

Professor Hyster highlighted the low substrate specificity of ketoreductases as a gateway to a diverse range of asymmetric photochemical transformations, including α-halocarbonyl reductions^46^ and intramolecular radical cyclizations.^47^ He also presented a broader range of asymmetric processes catalyzed by photoenzymes, including hydroamination reactions^48^ and even asymmetric sp^3^–sp^3^ cross-coupling reactions.^49^

His highly interdisciplinary approach beautifully reflected the thematic breadth and scientific diversity of this year's Bürgenstock Conference—an event that leaves a lasting impression.

## Final remarks

The 58th Bürgenstock Conference once again affirmed its reputation as a unique forum where scientific excellence meets intellectual generosity. Set against the serene backdrop of the Swiss Alps, this year's meeting offered more than just outstanding lectures, it fostered genuine dialogue across disciplines, career stages, and sectors.

From foundational discoveries to forward-looking technologies, the talks and discussions reflected the vibrant evolution of organic chemistry and its expanding frontiers. Yet what set the conference apart, as always, was not only the calibre of the science, but the spirit of exchange. As Nobel Laureate Roald Hoffmann once suggested, chemistry doesn't happen only in the lab – it thrives in conversation. At Bürgenstock, those conversations continue to shape the ideas, collaborations, and directions that will define the field for years to come.
